# Supplementary material for: Causal interplay between lactose intolerance and gut microbiota: a combined bidirectional Mendelian randomization and in vivo validation study
Source: Front Nutr. 2026 Jun 1;13:1803337. doi: 10.3389/fnut.2026.1803337 (PMC13265576; doi:10.3389/fnut.2026.1803337)
Supplement: Supplementary file 1 [file Data_Sheet_1.zip › supplementary materials/Forward/leave one out cross validation/ebi-a-GCST90027577.finngen_R12_E4_LACTONAS.pdf]

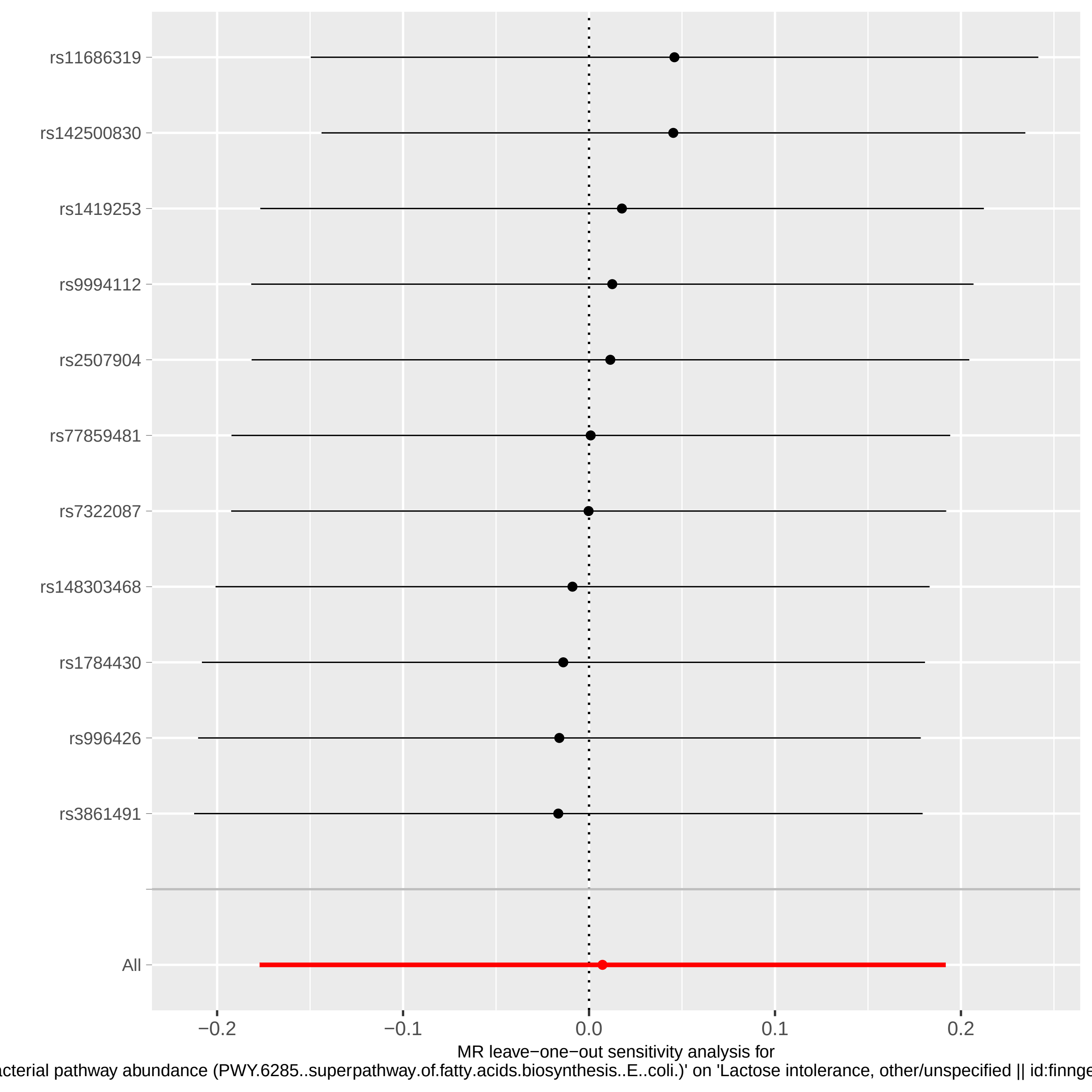
rs11686319
rs142500830
rs1419253
rs9994112
rs2507904
rs77859481
rs7322087
rs148303468
rs1784430
rs996426
rs3861491
All
−0.2
−0.1
0.0
0.1
0.2
MR leave−one−out sensitivity analysis for
acterial pathway abundance (PWY.6285..superpathway.of.fatty.acids.biosynthesis..E..coli.)' on 'Lactose intolerance, other/unspecified || id:finnge
